# Announcing the ISEV2020 special achievement award recipients: Andrew Hill and Edit Buzás; and the recipient of the ISEV2020 special education award: Carolina Soekmadji

**DOI:** 10.1002/jev2.12021

**Published:** 2020-11-05

**Authors:** Kenneth W. Witwer, Lucia R. Languino, Alissa M. Weaver, Marca H. Wauben

**Affiliations:** ^1^ Department of Molecular and Comparative Pathobiology The Johns Hopkins University School of Medicine Baltimore Maryland USA; ^2^ Department of Neurology The Johns Hopkins University School of Medicine Baltimore Maryland USA; ^3^ Sidney Kimmel Cancer Center Prostate Cancer Discovery and Development Program Thomas Jefferson University Philadelphia Pennsylvania USA; ^4^ Department of Cancer Biology Sidney Kimmel Cancer Center Thomas Jefferson University Philadelphia Pennsylvania USA; ^5^ Department of Cell and Developmental Biology Vanderbilt University School of Medicine Nashville Tennessee USA; ^6^ Program in Cancer Biology Vanderbilt University School of Medicine Nashville Tennessee USA; ^7^ Department of Pathology Microbiology and Immunology Vanderbilt University Medical Center Nashville Tennessee USA; ^8^ Department of Biochemistry and Cell Biology Faculty of Veterinary Medicine Utrecht University Utrecht The Netherlands

The International Society for Extracellular Vesicles (ISEV) is pleased to announce two Special Achievement Awards that were presented at the virtual ISEV2020 annual meeting, 20–22 July 2020. ISEV Special Achievement Awards are given each year to one or more individuals who have made outstanding contributions to the field of extracellular vesicle (EV) research or have performed extraordinary service to ISEV (Witwer, Hill, & Tahara, 2019). The ISEV special education award is given to an individual who made an outstanding contribution to drive the general education around EVs. In 2020, ISEV Special Achievement Awards were presented to Professors Andrew Hill and Edit Buzás. The ISEV education award was presented to Carolina Soekmadji (Figure 1).

**FIGURE 1:**
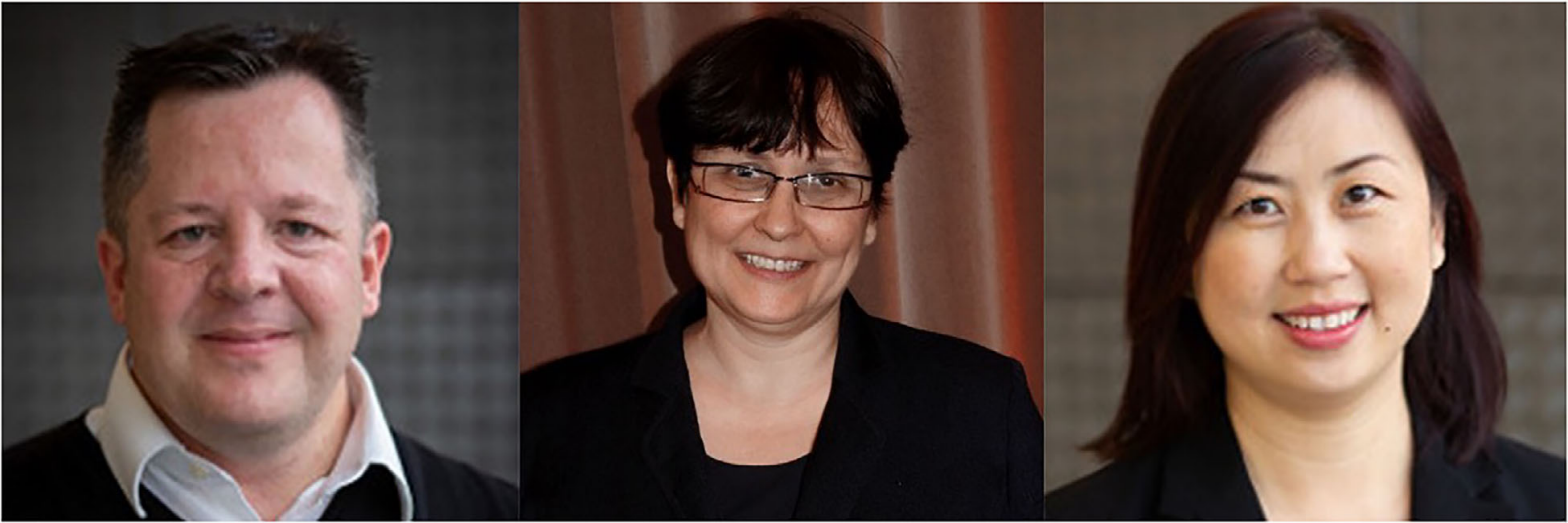
ISEV2020 Special Achievement and Special Education Award Recipients. Andrew F. Hill (left) and Edit I. Buzás (middle) received the ISEV2020 Special Achievement Awards. Carolina Soekmadji (right) was recipient of a Special Education Award.

## 1 ANDREW HILL

Andy Hill is Director of the La Trobe Institute for Molecular Science in Melbourne, Australia. He has been part of the ISEV board since the first elected board in 2012. During the first operating cycle of the ISEV board, Andy was a member‐at‐large, and during the second cycle, he became the first Executive Chair of Communication and Membership of ISEV. Thereafter, he was elected the second President of ISEV and served for two cycles as President. Under his leadership, ISEV matured into a true global scientific society, beginning a 3‐year rotation of annual meetings through all three chapters of ISEV: Asia/Pacific, Americas, and Europe/Africa. During his tenure, the society journal, the *Journal of Extracellular Vesicles*, also further developed into an established and highly respected journal, achieving indexing and journal impact factor tracking (Théry, Gho, & Quesenberry, 2019). With his modest, friendly and enthusiastic attitude, Andy has been a real bridge‐builder, embracing and fuelling all kinds of initiatives to promote and connect the EV field. He dedicated many hours—often not just during regular office time—and travelled enormous distances to lead the maturation of ISEV and the EV community. As President of ISEV, Andy actively reached out to other scientific societies to identify the needs and hurdles for clinical application of EVs. The importance of this outreach has become particularly clear in the time of COVID‐19 (Börger et al., 2020). He also recognized and promoted the importance of rigor and standardization in the EV field, leading to an update of MISEV (Théry et al., 2018) and the establishment of a Rigor and Standardization Subcommittee that has already been associated with several influential products (Clayton et al., 2019; Welsh et al., 2020).

For many years, Dr. Hill's research group has investigated EVs in neurodegenerative and inflammatory diseases. His group has made numerous valuable scientific contributions, ranging from basic principles of EVs in pathophysiological conditions to methodology development and potential clinical application of EV biomarkers and therapeutics. Examples include his contribution to our understanding of the role of EVs in prion disease (Guo, Bellingham, & Hill, 2015; Vella et al., 2007, 2008), a field in which Dr. Hill has made major strides for most of his career (Collinge, Sidle, Meads, Ironside, & Hill, 1996); the identification of circulating small non‐coding RNAs as biomarkers in neurodegenerative disease and rheumatoid arthritis (Cheng et al., 2015;, 2020; Bellingham, Coleman, & Hill, 2012; Foers et al., 2020); and the definition of crucial processing parameters to separate and study EVs of brain tissue (Huang et al., 2020; Vella et al., 2017).

In recognition of his extraordinary service to ISEV and to EV science and potential clinical application of EVs, an ISEV2020 Special Achievement Award was therefore presented to Professor Andrew F. Hill.

## 2 EDIT BUZÁS

Dr. Edit Buzás is Professor and Chair of the Department of Genetics, Cell‐ and Immunobiology at Semmelweis University in Budapest, Hungary. With a background in immunology, Edit has furthered our understanding of EV subtypes (Crescitelli et al., 2013; Osteikoetxea, Balogh, et al., 2015; Valcz et al., 2019), EVs in inflammation and cardiology (Giricz et al., 2014; Hegyesi et al., 2019; Marton et al., 2017; Osteikoetxea et al., 2016), and the roles that EVs play in the complex menagerie of blood and other biofluids, interacting with lipoproteins and protein complexes (Osteikoetxea, Sódar, et al., 2015; Sódar et al., 2016). Already in 2011, she published a highly influential review article on extracellular vesicles, emphasizing the diversity of EVs and endorsing “extracellular vesicle” as a collective term to encompass this diversity (Gyorgy et al., 2011). Edit is not only respected scientifically, but also beloved by her colleagues and mentees alike. She has been a prolific mentor to numerous scientists who have gone on to successful careers.

Dr. Buzás chaired the second ISEV Workshop (on isolation and characterization) in Budapest as well as the 2016 ISEV annual meeting in Rotterdam. She has provided crucial contributions to multiple ISEV position papers (Lener et al., 2015; Mateescu et al., 2017; Witwer et al., 2013). She served two terms as Executive Chair of Education, where she supervised the ISEV Education Days at the annual meetings and several workshops. Carrying on the work of previous Chair Yong Song Gho, two massive online open courses were released (Lässer et al., 2016) and another is now in planning, while a 3D animation, posters (Nieuwland et al., 2018), and other learning tools were developed. At the ISEV2020 General Assembly, she was confirmed as the 2020–2022 Secretary General of ISEV.

In an online interview, Edit advised those beginning EV studies to “be really open‐minded.” She said, “don't limit your imagination if you have a brave idea. In this novel field, you have a chance to find something really prominent” (Buzás, 2020). Introducing her as an award winner during ISEV2020, Kenneth Witwer stated, “Edit should know, as someone who has made and continues to make ‘really prominent’ contributions.”

For her major contributions to the EV field and to ISEV, Professor Edit I. Buzás was a 2020 recipient of the ISEV Special Achievement Award.

## 3 SPECIAL EDUCATION AWARD: CAROLINA SOEKMADJI

At ISEV2020, a Special Education Award was announced for only the second time in ISEV history. The first ISEV massive online open course (MOOC), on the Basics of Extracellular Vesicles (Lässer et al., 2016), was supervised by Cecilia Lässer, who received the first Special Education Award in 2016. Following the success of MOOC I, Carolina Soekmadji volunteered to coordinate a second MOOC, on EVs in Health and Disease. Dr. Soekmadji is a Senior Research Officer at Queensland Institute of Medical Research, Berghofer, in Brisbane, Australia, and she has served as a Member‐at‐Large and an Adjunct Member of the ISEV Board. She has also contributed to several ISEV education and standardization initiatives (Soekmadji et al., 2018, Witwer et al., 2017). MOOC II provides 10 hours of content to learners and had attracted almost 2000 registrants by ISEV2020. For her outstanding contributions to MOOC II, Carolina Soekmadji was presented with the ISEV2020 Special Education Award.
